# The Effect of Light Exposure on Water Sorption and Solubility of Self-Adhesive Resin Cements

**DOI:** 10.1155/2014/610452

**Published:** 2014-10-29

**Authors:** Thaiane Rodrigues Aguiar, Carolina Bosso André, Gláucia Maria Boni Ambrosano, Marcelo Giannini

**Affiliations:** ^1^Department of Restorative Dentistry, Piracicaba Dental School, State University of Campinas, Avenue Limeira 901, Bairro Areião, 13414-903 Piracicaba, SP, Brazil; ^2^Department of Social Dentistry, Piracicaba Dental School, State University of Campinas, Avenue Limeira 901, Bairro Areião, 13414-903 Piracicaba, SP, Brazil

## Abstract

*Purpose.* To investigate the effect of light activation on the water sorption (WS) and solubility (SL) of resin cements after 24 h and 7 days. *Methods*. Disk-shaped specimens were prepared using five dual-polymerized cements (four self-adhesive [RelyX Unicem, MaxCem, SeT and G-Cem] and one conventional [Panavia F 2.0]) and divided according to the curing mode (direct light exposure or self-cure) and water immersion period (24 h or 7 days). Specimens were dry-stored and weighed daily until a constant mass was recorded (M1). Then, specimens were stored in water for either 24 h or 7 days and immediately weighed (M2). After desiccation, specimens were weighed again until a constant mass was achieved (M3). WS and SL were calculated and statistically analyzed by Kruskal-Wallis, Dunn and Mann-Whitney *U* tests (*α* = 0.05%). *Results.* There was a significant increase in WS for all products after one-week immersion in water. The highest water uptake was observed for autopolymerized groups. Extended water immersion significantly affected the SL for most of autopolymerized cements. Significant differences between products were observed in both tests. *Conclusions.* The curing mode and the water immersion period may affect the mechanical stability of the resin cements, and these differences appear to be product-dependent.

## 1. Introduction

The development of self-adhesive resin cements changed the clinical concept of adhesive cementation. These materials targeted to simplify the clinical procedures by eliminating critical steps such as the phosphoric acid etching and bonding application. The bonding mechanism of self-adhesive resin cements is based on the presence of acidic monomers, and neither resin tags nor conventional hybrid layers were observed in the adhesive interface [1–4]. Interestingly, immediate and long-term dentin bonding studies demonstrated that the self-adhesive cements seem to be a good alternative for indirect restorations and fiber posts cementation [5–8]. On the other hand, the lack of information about these new materials impairs better understanding of their biomechanical behavior.

Adequate high-density network polymer formation plays a crucial role in the mechanical properties of resin-based materials. The photoactivation of dual-cured resin cements increased the degree of conversion and led to better clinical outcomes [9–13]. However, in some clinical situations, the absence or reduction of light intensity may affect the resistance to polymer degradation and then compromise their mechanical properties. The presence of residual unreacted monomers acts as a plasticizer and may increase the water uptake, ions diffusion, and solubility of resin compounds [14–17]. The water sorption and solubility properties of dental resin-based materials are also associated with the composition of the materials, including the hydrophilic nature of resin monomers, type and concentration of solvents, and inorganic composition [15–18].

The water sorption and solubility of adhesive systems and resin composites have been widely investigated [16–21]; however, fewer studies assessed these properties of self-adhesive resin cements [22–24]. Moreover, to the best of our knowledge, limited information is available regarding the effect of curing mode on the gain/loss of mass of self-adhesive agents. Thus, the aim of this study was to evaluate the effect of curing mode and water storage time on the water sorption and solubility of five resin cements (one conventional and four self-adhesive materials). The null hypothesis tested was that the curing mode and water immersion period would not affect the water sorption and solubility of resin cements.

## 2. Materials and Methods

### 2.1. Specimen Preparation

Water sorption and solubility tests were performed in compliance with the International Organization for Standardization-Dentistry-Polymer-based filling, restorative and luting materials (ISO 4049:2000). Five dual-cured resin cements were tested: one conventional (Panavia F 2.0/PF) and four self-adhesive resin cements (RelyX Unicem/RU, MaxCem/MX, SeT/ST, and G-Cem/GC). The manufacturer's composition and lot number of the resin cements are listed in Table 1. Sixteen disk-shaped specimens (6.0 ± 0.1 mm diameter  ×  1.0 ± 0.1 mm thickness) were prepared for each material and then divided into 4 groups (*n* = 4 per group) according to curing mode (light activated or autopolymerized) and water immersion period (24 h or 7 days). The commercial resin cements were manipulated according to the manufacturers' instructions and placed in a circular silicone mold (Aquasil Ultra LV, Dentsply Caulk, Milford, DE, USA). Then, the surface of each specimen was covered with a Mylar strip to avoid oxygen inhibition, prevent air bubbles, and create a flat surface [24].

Specimens were let them either self-cure or irradiated with a Quartz Tungsten Halogen based curing unit for 40 seconds (XL 3000, 3M ESPE, St. Paul, MN, USA), and immediately stored in dark container at 37°C for 24 h. The light intensity was 600 mW/cm^2^ and the output irradiance was measured with a radiometer unit (Model 100, Kerr Corp, Orange, CA, USA). Then, specimens were carefully removed from their molds and the excess was removed using number 1000 grit silicon carbide paper. In order to calculate the volume (V) of each specimen in cubic millimeters, specimen's dimensions were measured using a digital micrometer (Mitutoyo Sul Americana Ltda, Suzano, SP, Brazil).

### 2.2. Water Sorption and Solubility

Specimens were placed in a desiccator at 37°C and weighed daily in an analytical balance (JK-180, Chiyo Balance Corp., Tokyo, Japan) until a constant mass (M1) was recorded (i.e., until the mass loss of each specimen was not more than 0.1 mg within a period of 24 h). Afterwards, specimens were individually stored in distilled water for 24 h or 1 week. Following water immersion, disk-shaped specimens were washed with distilled water, blotted quickly using soft absorbent paper, and weighed again (M2). Then, specimens were dry-stored and the mass was daily recorded until a constant mass was obtained as described before (M3). The values of water sorption (WS) and solubility (SL) were calculated in *μ*g/mm^3^ using the following equations:

(1) $$\begin{array}{c} \text{WS}=\frac{\text{M}2-\text{M}3}{\text{V}}\;\;\text{SL}=\frac{\text{M}1-\text{M}3}{\text{V}}, \end{array}$$

where M1 is the conditioned mass (*μ*g) prior to immersion in water; M2 is the mass of the specimen (*μ*g) after immersion in water for 24 h or 1 week; M3 is the mass of the reconditioned specimen (*μ*g); V is the volume of each specimen (mm^3^).

### 2.3. Statistical Analysis

The nonparametric data of the water sorption and solubility were statistically analyzed by Kruskal-Wallis, Dunn, and Mann-Whitney tests, with a 95% confidence level.

## 3. Results

Water sorption and solubility results are summarized in Tables 2 and 3, respectively. WS revealed statistically significant differences for the factors “resin cement,” “storage time,” and “curing mode” (*P* < 0.05). After 24 h, a significant increase in WS was observed for PF, RU, and SeT resin cements in autopolymerized mode when compared to photopolymerized condition. In addition, the water sorption significantly increased when specimens were kept in water for one week in both curing conditions (light activated and autopolymerized cements); however, autopolymerized products demonstrated higher WS than the light activated resin cements. Regarding the light activated mode, Panavia F and RelyX Unicem exhibited significant lower WS after immersion in water during 24 h and 7 days.

According to the solubility analysis, there were statistically significant differences among all factors studied (*P* < 0.05). Regarding the curing mode, significant differences on SL were observed for Panavia F and MaxCem after 24 h water storage. Light activated resin cements demonstrated lower SL when compared to autopolymerized resin cement after 7 days in water; only G-Cem was not affected by the curing mode. Overall, significant differences were observed between products under the same condition, showing changes in the WS and SL regardless of the water storage time or curing mode.

## 4. Discussion

The long-term clinical performance of resin-based materials has been associated with many factors, which includes the ability of material to uptake/dissolve in water. This investigation demonstrated that the curing mode and water storage period affected the water sorption and solubility of resin cements. Therefore, the hypothesis tested was rejected.

The hydrophilicity of polymeric matrices and the inorganic composition of resin cements may affect the water sorption and solubility behavior [14–18, 25–27]. Interestingly, liquid water exposure of thermosetting systems has been described to induce weight gain and weight losses, and such diffusion phenomenon is much more slower due to size of the diffusing molecules [28, 29]. In this study, the water sorption of resin cements tested ranged from 9.0 to 112.2 *μ*g/mm^3^. Some values were higher than those required by the ISO 4049 standard, which establishes that the maximum water sorption value is 40 *μ*g/mm^3^. It appeared to be associated with changes of the specimen's dimensions and the curing mode when compared to the method outlined in ISO 4049. However, the water sorption means obtained for light-cured specimens were similar to those obtained by Gerdolle et al. [23] (17.9 and 21.6 *μ*g/mm^3^) and Marghalani [24] (15.1 to 23.1 *μ*g/mm^3^ after 24 h and 20.0 to 35.1 *μ*g/mm^3^ after 1 week).

The light exposure affected the water sorption and solubility of the resin cements. After 7 days of being immersed in water, the autopolymerized specimens showed highest water uptake and solubility for most of products tested. It appeared to be related to the lower degree of conversion of autopolymerized resin cements when compared to light-activated resin-based materials [10, 30]. In accordance with these findings, Moreira et al. [31] demonstrated that the light unit source and the curing regimen may influence the water sorption. Interestingly, the weight losses were observed for some specimens and it seems to be affected by the kinetic of water uptake of each material.

Considering the curing mode, Maxcem showed similar water sorption in each water immersion period. Maxcem self-adhesive resin cement contains multifunctional DMAs and GPDM, barium, fluoroaluminosilicate, and fumed silica filler particles according to the manufacturer's information. The high number of fillers (66 wt.%) apparently reduces the organic content of this product, which may change some physical properties of resin-based materials, and explains the water sorption data observed in this study.

Regarding solubility, there were few significant differences among resin cements in both water storage time and curing mode conditions. All materials showed at least one significant difference in solubility when compared to the water immersion periods (24 h or 7 days). The negative solubility values demonstrated for SeT and G-Cem resin cements suggested that the absorbed water was not completely eliminated by the drying process or the curing reaction was not completed, such as the self-curing. Curiously, Panavia F 2.0 resin cement was the only one to show increased solubility when self-cured in both storage times.

Panavia F 2.0 is categorized as traditional resin cement and was used as control group since other resin cements tested are classified as self-adhesive materials. The Panavia F 2.0 contains 10-MDP as functional monomer; filler particles composed of silica and barium glass and hydrophobic aromatic dimethacrylate, hydrophobic aliphatic methacrylated, and hydrophilic aliphatic dimethacrylate monomers to form a highly cross-linked cement matrix after polymerization. In the current study, Panavia F 2.0 was not mixed with ED Primer, as happening in clinical scenario. According to the manufacturer, ED primer is applied to tooth surface and the resin cement (paste A and B mixed) must be applied to the prosthesis or postthesis. The contact between ED Primer and resin cement accelerates the autopolymerization reaction of this material. Thus, a significant increase of solubility and water sorption values in autopolymerization mode obtained herein may be due to the absence of the bonding agent. ED Primer improves the degree of conversion of Panavia F 2.0 and reduces the amount of unreacted monomers that can be soluble in water [9, 32].

RelyX Unicem self-adhesive resin cement contains dimethacrylate-ester of phosphoric acid, which has an ability to interact with tooth surface. Calcium hydroxide is the alkaline portion that neutralizes the remaining phosphoric acid groups and becomes the material more hydrophobic, less water sorption, and soluble. The fluoride releasing is achieved in the course of the cement setting reaction by ionization of glass powders. The amount of inorganic fillers contained in RelyX Unicem is approximated to be 70% by weight (information supplied by the manufacturer) and revealed irregular-shaped particles with approximately 0.5 *μ*g in size [33]. Since the characteristics and percentage of filler particles can vary among materials studied, this factor may affect the findings obtained in this study.

SeT and G-Cem self-adhesive resin cements also present fluoride-releasing properties due to the presence of fluoro-alumino-silicate glass. Both materials contain acidic functional monomers and UDMA; however, the 4-META monomer is incorporated only into the G-Cem resin cement. 4-META is an acidic monomer that interacts with the tooth substrate. The molecule contains two functional groups: a hydrophilic and another hydrophobic group [34], while UDMA resin is hydrophobic polymer with low water sorption and solubility [35].

The evaluation of self-adhesive resin cements was the main focus of this study; self-adhesive materials showed few significant differences when compared to traditional resin cement. Overall, resin cements that showed an increase of water sorption/solubility may present early hydrolytic degradation, which reduces the lifetime of restorations. The self-adhesive resin cements contain functional monomers that form bonds with calcium or other structures of the tooth, promoting the adhesion to the dentin surface. Specific composition and concentrations of each component are generally not available for all materials studied. This information is crucial to discuss the findings showed herein since the presence of more hydrophilic monomers, ionic resin monomers, and/or other products may directly influence the water sorption and solubility behavior of these resin-based materials.

## 5. Conclusion

The water sorption and solubility of the resin cements appeared to be even more critical under self-cured condition. Thus, photo-activation may prevent the hydrolytic degradation throughout the lifetime of indirect restorations.

## Figures and Tables

**Table 1:** The manufactures' compositions and lot number of the dual-cured resin cements used in this investigation.

Resin cement (Manufacturer)	Composition (Lot number)
G-Cem (GC Corp., Tokyo, Japan)	4-META, UDMA, fluoro-alumino-silicate glass, pigment, DMA, distilled water, phosphoric ester monomer, initiator, and camphorquinone (0702231).
MaxCem (Kerr Corp., Orange, CA, USA)	Multifunctional DMAs, GPDM, proprietary Redox initiators and photo-initiators, barium, fluoroaluminosilicate, and fumed silica (66 wt.%) (2741040).
Panavia F 2.0 (Kuraray Medical Inc., Kurashiki, Okayama, Japan)	Paste A: 10-MDP, hydrophobic and hydrophilic DMA, silanized silica filler, silanated colloidal silica, dl-camphorquinone, initiators, and others (00249A). Paste B: hydrophobic and hydrophilic DMA, sodium fluoride, silanated barium glass filler, initiators, accelerators, and pigments (00026B).
RelyX Unicem (capsules) (3M ESPE, Seefeld, Germany)	Powder: silanized glass powder (85–95% wt.), silane treated silica (5–10% wt.), substituted pyrimidine (1–5% wt.), calcium hydroxide (<3% wt.), and sodium persulfate (<1% wt.). Liquid: dimethacrylate-ester of phosphoric acid (40–50% wt.), TEGDMA (25–35% wt.), and substituted dimethacrylate (20–30% wt.) (279915).
SeT (SDI, Bayswater, VIC, Australia)	UDMA; fluoroaluminosilicate glass (60–70 wt.%); camphorquinone (<1 wt.%), and acidic monomer (<20 wt.%) (50711272).

Abbreviations: 10-MDP: 10-methacryloyloxydecyl dihydrogen phosphate; DMA: dimethacrylates; TEGDMA: triethylene glycol dimethacrylate; GPDM: glycerol dimethacrylate dihydrogen phosphate; Bis-GMA: bisphenol-A-diglycidyl ether dimethacrylate; UDMA: urethane dimethacrylate; 4-META: 4-methacryloxyethyl trimellitate anhydride.

**Table 2:** Median (minimum value; maximum value) of water sorption (*µ*g/mm^3^) for resin cements according to water storage time and curing mode.

Resin cement	Water immersion—24 h		Water immersion—7 days	
	Light activated	Autopolymerized	Light activated	Autopolymerized
Panavia F 2.0	10.0 (9.4; 10.1) Bb	23.0 (13.0; 38.3) Aab	35.1 (31.6; 49.6) Bab^*^	112.2 (101.1; 147.6) Aa^*^
RelyX Unicem	9.0 (3.6; 11.0) Bb	18.1 (16.7; 24.6) Ab	32.0 (27.2; 49.2) Bb^*^	70.0 (53.2; 92.0) Aab^*^
Maxcem	32.4 (26.6; 34.8) Aa	29.1 (28.5; 30.2) Aab	73.4 (69.9; 85.4) Aa^*^	79.3 (63.2; 108.1) Aab^*^
SeT	31.9 (28.7; 33.6) Ba	39.3 (38.5; 42.0) Aa	81.7 (79.7; 95.6) Aa^*^	91.2 (87.2; 94.2) Aab^*^
G-Cem	28.1 (25.2; 33.7) Aa	27.3 (25.8; 31.5) Aab	36.3 (35.1; 40.1) Bab^*^	52.1 (50.9; 53.6) Ab^*^

Different letters and symbols represent statistical significance among the factors studies (*P* ≤ 0.05).

Asterisks (∗) compare the water immersion times (24 h and 7 days) within each resin cement and curing mode.

Capital letters compare the curing mode factor within each water immersion time.

Lower case letters compare resin cements irrespective of curing mode and water immersion time.

**Table 3:** Median (minimum value; maximum value) of solubility (*µ*g/mm^3^) for resin cements according to water storage time and curing mode.

Resin cement	Water immersion—24 h		Water immersion—7 days	
	Light activated	Autopolymerized	Light activated	Autopolymerized
Panavia F 2.0	4.9 (3.4; 6.7) Bab	10.9 (6.5; 20.7) Aa	9.7 (6.2; 15.5) Ba	30.9 (21.7; 47.2) Aa^*^
RelyX Unicem	3.6 (0.0; 3.6) Aab	3.2 (0.0; 6.9) Aab	0.0 (0.0; 3.4) Bab	32.6 (15.6; 41.3) Aa^*^
Maxcem	7.2 (3.8; 11.0) Aa	3.6 (0.0; 3.7) Bab	13.0 (3.1; 19.1) Ba	29.6 (20.6; 37.2) Aa^*^
SeT	−1.6 (−3.4; 0.0) Ab	−1.6 (−6.6; 3.4) Ab	−8.3 (−11,8; −6.4) Bb^*^	−3.2 (−6.4; −3.0) Ab
G-Cem	5.5 (−3.5; 9.4) Aab	3.1 (0.0; 6.3) Aab	−4.9 (−7.0; −3.1) Aab	−1.7 (−3.4; 0.0) Ab^*^

Different letters and symbols represent statistical significance among the factors studied (*P* ≤ 0.05).

Asterisks (∗) compare the water immersion time (24 h and 7 days) within each resin cement and curing mode.

Capital letters compare the curing mode factor within each water immersion time.

Lower case letters compare resin cements irrespective of curing mode and water immersion time.
